# SnRK1α1 Antagonizes Cell Death Induced by Transient Overexpression of *Arabidopsis thaliana* ABI5 Binding Protein 2 (AFP2)

**DOI:** 10.3389/fpls.2020.582208

**Published:** 2020-09-29

**Authors:** Carina Steliana Carianopol, Sonia Gazzarrini

**Affiliations:** ^1^Department of Biological Sciences, University of Toronto Scarborough, Toronto, ON, Canada; ^2^Department of Cell and Systems Biology, University of Toronto, Toronto, ON, Canada

**Keywords:** SnRK1, AFP2, abscisic acid, cell death, programmed cell death

## Abstract

Plants are continuously exposed to environmental stressors. They have thus evolved complex signaling pathways to govern responses to a variety of stimuli. The hormone abscisic acid (ABA) has been implicated in modulating both abiotic and biotic stress responses in plants. ABI5 Binding Proteins (AFPs) are a family of negative regulators of bZIP transcription factors of the AREB/ABF family, which promote ABA responses. AFP2 interacts with Snf1-Related protein Kinase 1 (SnRK1), which belongs to a highly conserved heterotrimeric kinase complex that is activated to re-establish energy homeostasis following stress. However, the role of this interaction is currently unknown. Here, we show that transient overexpression of *Arabidopsis thaliana AFP2* in *Nicotiana benthamiana* leaves induces cell death (CD). Using truncated AFP2 constructs, we demonstrate that CD induction by AFP2 is dependent on the EAR domain. Co-expression of the catalytic subunit SnRK1α1, but not SnRK1α2, rescues AFP2-induced CD. Overexpression of SnRK1α1 has little effect on AFP2 protein level and does not affect AFP2 subcellular localization. Our results show that a high level of AFP2 is detrimental for cell function and that SnRK1α1 antagonizes AFP2-induced CD most likely through a mechanism that does not involve AFP2 protein degradation or a change in subcellular localization.

## Introduction

The hormone abscisic acid (ABA) plays an important role in plant development and stress response. (Cutler et al., 2010; Raghavendra et al., 2010; Finkelstein, 2013; Vishwakarma et al., 2017; Yoshida et al., 2019). In the presence of ABA, Pyrabactin Resistance (PYR)/PYR-like (PYL)/Regulatory Component of ABA Receptor (RCAR) receptors inhibit clade A protein phosphatase 2C (PP2C) activity. In the absence of PP2Cs, Snf1-related protein kinase2 (SnRK2) become activated and phosphorylate downstream targets, including transcription factors (TFs) and anion channels, leading to ABA-regulated gene expression and adaptive response such as germination and growth inhibition, as well as stomata closure and leaf senescence (Cutler et al., 2010; Raghavendra et al., 2010). Basic leucine zipper (bZIP) TFs such as Abscisic Acid Insensitive5 (ABI5), ABA-Response-Element Binding1 (AREB1)/ABRE-binding factors (ABF2) and AREB2/ABF4, which are SnRK2 phosphorylation targets, bind the ABA response element (ABRE) in the promoter of target genes and induce ABA-dependent transcriptional changes (Furihata et al., 2006; Fujii et al., 2007; Fujita et al., 2009; Cutler et al., 2010; Finkelstein, 2013; Yoshida et al., 2015; Zhu, 2016).

ABI5/ABF are also phosphorylated by SnRK1 and SnRK3 kinases (Bitrián et al., 2011; Zhou et al., 2015). SnRK1 is a highly conserved heterotrimeric kinase complex, which is involved in reprogramming gene expression to adjust growth and development during stress response in eukaryotes (Broeckx et al., 2016; Wurzinger et al., 2018; Crepin and Rolland, 2019). The catalytic subunit of the SnRK1 complex, SnRK1α, is positively regulated by ABA. Similar to SnRK2s, ABA prevents SnRK1 dephosphorylation, and thus its deactivation, through sequestration of PP2C phosphatases (Rodrigues et al., 2013). Activation of SnRK1 or ABA signaling results in the induction of a common set of transcriptional targets, suggesting that these two signaling pathways may regulate common target genes during stress (Rodrigues et al., 2013).

To identify common targets of the ABA and SnRK1 signaling pathways, we conducted a high-troughput yeast two-hybrid (Y2H) screen and showed that the SnRK1 complex interacts with 125 ABA-regulated proteins, including both positive and negative regulators of ABA signaling, suggesting that the SnRK1 and ABA signaling pathways largely intersect during plant stress responses (Carianopol et al., 2020). One of the interactors identified is ABI Five Binding Protein2 (AFP2), which belongs to a subfamily of four negative regulators of ABI5/ABFs; these AFPs are members of the Novel Interactor of JAZ (NINJA) and NINJA-like superfamily of adaptor proteins (Lopez-Molina et al., 2003; Garcia et al., 2008; Lynch et al., 2017). In the absence of stress, AFPs attenuate the ABA response. The EAR (ethylene-responsive element binding factor-associated amphiphilic repression) domain of AFP2, AFP3, and NINJA proteins bind the co-repressor TOPLESS (TPL), which represses expression of target genes by interacting with histone deacetylase (HDAC) complexes (Pauwels et al., 2010; Causier et al., 2012; Liu et al., 2014; Lynch et al., 2017; Martin-Arevalillo et al., 2017; Chang et al., 2019). This mechanism is conserved in monocots (Tang et al., 2016).

Despite the important role of AFPs in attenuating the ABA response, the mechanism regulating AFP function in the absence or presence of stress is unknown. Here, we aimed to understand the role of SnRK1 interaction with AFP2. We show that transient overexpression of Arabidopsis *AFP2* is sufficient to induce cell death (CD) in *Nicotiana benthamiana* leaves, suggesting that increased expression of *AFP2* has a detrimental effect on cell function. By generating AFP2 truncation variants, we show that the EAR and B domains of AFP2 are necessary and sufficient for induction of CD, suggesting that AFP2 recruitment of TPL/HDACs through the EAR domain may play a role in AFP2-induced CD. SnRK1α1, but not SnRK1α2, can rescue AFP2-mediated CD induction when both proteins are transiently co-expressed. Thus, SnRK1 can directly or indirectly counteract the AFP2-mediated induction of CD. This study uncovers novel and contrasting roles for AFP2 and SnRK1α1 during CD.

## Materials and Methods

### Plant Material and Growth Conditions

*Nicotiana benthamiana* seeds were directly germinated on soil (PromixPGS; Plant Products) and grown under long day conditions (16 h of light at 28°C and 8 h of darkness at 24°C). Seedlings were transferred to individual pots 10 days after germination and grown in controlled growth chambers (Conviron) under long day conditions.

### Yeast Two Hybrid

AFP2 and its truncations were cloned in the bait vector (pEZY202), fused to the LexA DNA binding domain (DBD) and transformed into *Saccharomyces cerevisiae* strain RFY206a (MATa) harboring the *LacZ* reporter (pSH18-34 plasmid). SnRK1α1 was cloned in the prey vector (pEZY45), fused to the B42 activation domain (AD) and transformed into *S. cerevisiae* strain EGY48α (MATα) carrying the chromosomal *LEU* reporter. All cloning was performed through recombination using Gateway cloning system (Invitrogen). Yeast cells were mated on selective media lacking leucine (YNB ura- his- trp- leu- supplemented with galactose and raffinose) or supplemented with 0.1 mg/ml X-gal (YNB ura- his- trp- supplemented with glucose) and interactions were scored by identifying colony growth on leu- plates and blue colonies on X-gal plates, respectively. The mating protocol was performed as described in Clontech Yeast Protocol Handbook (2011). None of the bait constructs auto-activated the reporters. Pictures were taken after 4 days of growth on selection media. Each mating was performed twice on each reporter, and the same results were obtained.

### Bimolecular Fluorescence Complementation

Interactions between SnRK1α1 and α2 with AFP2 were tested in *N. benthamiana* leaves by Bimolecular Fluorescence Complementation (BiFC) assays. Half-YFP (YFP^N^/YFP^C^) fusion constructs were cloned in pB7WGYn2 and pB7WGYc2 vectors (Lumba et al., 2014). All constructs were transformed into *Agrobacterium tumefaciens* strain GV2260. The transient BiFC assays were performed using four-week old *N. benthamiana* plants grown under long days (16 h of light at 28°C and 8 h of darkness at 24°C). The abaxial side of the leaf was syringe-infiltrated with the infiltration media (1% yeast extract, 1% peptone, 0.5% sodium chloride), which contained *Agrobacterium* carrying CaMV35S::HC-Pro from tobacco etch virus (TEV) to suppress gene silencing at a final OD_600_ of 0.2 and 150 μM acetosyringone (Lewis et al., 2008). For each biological replicate, three leaves were infiltrated. As a control, *N. benthamiana* was infiltrated with the infiltration media only. Following infiltration, plants were grown under short days (8 h of light at 22°C, 16 h of darkness at 22°C) for 72 h. Fluorescence was visualized using an LSM550 confocal microscope (Zeiss). Confocal images were taken with a 514 nm excitation laser, with a 515–535 nm bandpass filter for YFP emission. Autofluorescence from chlorophyll was detected at ≥585 nm. Plant tissue was directly mounted on glass slides in double distilled water. Each interaction was detected at least in three biological replicates, with three leaves infiltrated each time. Interactions were observed in multiple cells, with a frequency of at least 1 in 10 cells. At least 100 cells per leaf disk were analyzed each time; the same results were obtained in each biological replicate and one representative image is shown.

### Quantification of Cell Death

Quantification of cell death (CD) was done six days post infiltration of *N. benthamiana* leaves, using a plant immunity and disease image-based quantification (PIDIQ) method, whose results are comparable to conducting traditional assays such as ion leakage and *in planta* pathogen growth assays (Laflamme et al., 2016). Briefly, an image of each *N. benthamiana* half-leaf was taken and processed by PIDIQ macro in ImageJ, which quantifies healthy (green) and unhealthy (grey) tissue (**Supplemental Figure S1**). The following changes in program specifications were implemented to adjust for usage with *N. benthamiana* instead of *A. thaliana*, and grey was used rather than yellow for unhealthy tissue: green area (healthy) conditions: “Hue” min =25, max = 104; “Saturation” min = 0, max = 255; “Brightness” min = 65, max = 255 and CD area (unhealthy) conditions: “Hue” min = 25, max = 50; “Saturation” min = 0, max = 92; “Brightness” min = 80, max = 255. A ratio of unhealthy tissue versus total tissue was then calculated by measuring the total number of pixels of unhealthy tissue (grey) divided by the sum of pixels of total infiltrated tissue [unhealthy (grey) plus healthy (green) tissue]. Three infiltrated leaves per plant per construct were used in each trial and the experiment was repeated at least twice. The averages of three biological replicates with standard error of the mean (SEM) are shown in each graph. Statistical analysis was performed using one-way ANOVA, followed by Tukey HSD multiple comparisons of means in R.

### Protein Extraction and Immunoblotting

*N. benthamiana* leaf discs were first checked for protein expression by confocal microscopy (localization of YFP-protein fusion or BiFC interaction). Proteins were then crude extracted from microscopy-confirmed *N. benthamiana* leaves at three DAI, by grinding 50 mg of flash frozen leaf discs in 200 ul of 2x SDS-Laemmli buffer (278 mM Tris-HCl pH 6.8, 44.4% glycerol, 4.4% SDS, 0.02% bromophenol blue). The HA-tagged proteins were detected using 1:1,000 Rabbit anti-HA primary antibody (Cedarlane) and 1:5,000 Peroxidase-AffiniPure Donkey Anti-Rabbit secondary antibody (Jackson ImmunoResearch). The YFP^C^-AFP2 protein was detected using 1:2,000 purified Mouse anti-myc primary antibody (Cedarlane) and 1:5,000 Peroxidase-AffiniPure Goat Anti-Mouse (Cedarlane) secondary antibody. The YFP-fused proteins were detected using 1:5,000 purified Rabbit anti-GFP primary antibody (Abcam) and 1:5,000 Peroxidase-AffiniPure Goat Anti-Mouse secondary antibody (Cedarlane). Pierce™ ECL (Thermofisher) Western blotting substrate (HRP substrate for enhanced chemiluminescence) was used for protein detection. At least three biological replicates were performed, and one representative blot is shown. Quantification of immunoblots was done using ImageJ by first measuring pixel values of the protein bands in an equal area and normalizing them to the pixel values of the Rubisco large subunit band on the Ponceau blot. Then, the relative protein level was calculated as a ratio to the normalized empty vectors (EV) control band intensity. The EV controls were set to one, and all sample values are given relative to the control (Chiu et al., 2016). Quantification of three biological replicates was performed.

## Results

### SnRK1α1 Inhibits AFP2-Mediated Induction of Cell Death in *N. benthamiana*

**Figure 1:**
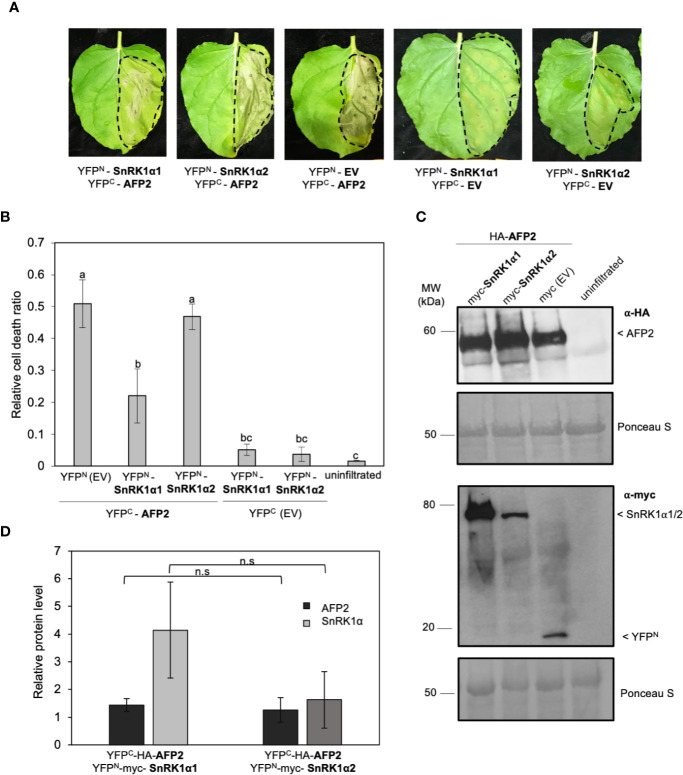
SnRK1α1, but not SnRK1α2, negatively regulates cell death induced by ectopic overexpression of *AFP2* in *N. benthamiana* leaves. **(A)** Leaf phenotype observed six days after infiltration (DAI) of *N. benthamiana* leaves with YFP^C^-HA-AFP2 and YFP^N^-myc-SnRK1α. EV, empty vector (YFP^C^-HA or YFP^N^-myc). Dashed line delineates the infiltration area. **(B)** Relative quantification of cell death (dead tissue versus total infiltrated tissue) in *N. benthamiana* leaves at six DAI using ImageJ macro PIDIQ (Laflamme et al., 2016). Average of three biological replicates ± SEM is shown; p < 0.05, ANOVA and *post hoc* Tukey HSD test. **(C)** Immunoblot showing YFP^C^-HA-AFP2 (58 KDa) and YFP^N^-myc-SnRK1α1/YFP^N^-myc-SnRK1α2 (76 kDa/79 kDa) protein levels three DAI in *N. benthamiana* leaves, using anti-HA or anti-myc antibody. Ponceau stain is shown as loading control. **(D)** Quantification of AFP2 (YFP^C^-HA-AFP2) and SnRK1a (YFP^N^-myc-SnRK1α) protein levels relative to the respective EV controls (the EV was set to 1). Values were normalized to the Rubisco large subunit in the Ponceau stain. Average of three biological replicates ± SEM is shown; p > 0.05, two-way Student’s t-test. Small letters represent the statistical grouping based on ANOVA and post hoc Tukey HSD test.

We have previously shown that AFP2 interacts with the SnRK1 catalytic subunits, SnRKα1 and SnRKα2, in Y2H and BiFC assays (Carianopol et al., 2020). While transiently expressing AFP2 with the empty vector control in *N. benthamiana*, we observed that AFP2 induced CD in the infiltrated area of the leaf (**Figures 1A, B**; **Supplemental Figure 1**). The CD phenotype was visible three to four days post infiltration, and clearly recognizable six days post infiltration. The leaf tissue turned grey and dried, but CD was limited to the infiltrated area and did not spread to nearby tissues (**Figure 1A**). However, when AFP2 was co-infiltrated with SnRK1α1, a strong reduction of CD was observed compared to leaves that were infiltrated solely with AFP2 and the empty vector control (**Figures 1A, B**; **Supplemental Figure 1**; p < 0.01, ANOVA and *post hoc* Tukey HSD test). Neither SnRK1α1 nor α2 were able to induce CD on their own (**Figures 1A, B**; **Supplemental Figure 1**). Although not statistically significant, a slight CD reduction could also be seen in leaves co-expressing AFP2 and SnRK1α2 (**Figure 1B**).

We then tested whether the reduction in CD was due to a reduction in AFP2 protein level. The results show that AFP2 protein levels were not affected by co-infiltration with either of the SnRK1α subunits (**Figures 1C, D**; ANOVA p > 0.05). However, the average level of SnRK1α1 protein is slightly higher than that of SnRK1α2, possibly explaining the weaker rescue of CD by SnRK1α2. This data suggests that SnRK1α1 can antagonize CD induced by transient overexpression of AFP2 *in planta*, and that the SnRK1α1-mediated CD rescue is not due to a reduction in AFP2 protein level.

### Conserved Domains of AFP2 Localize to the Nucleus *In Planta*

**Figure 2:**
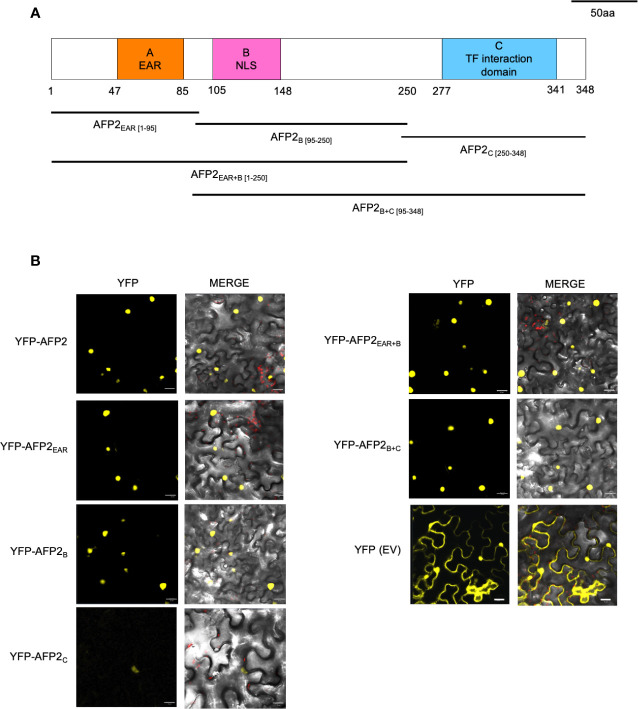
Subcellular localization of AFP2 truncations in *N. benthamiana* leaves. **(A)** Schematic of the AFP2 protein showing the conserved A, B and C domains (Garcia et al., 2008). Drawing is done to scale, and numbers correspond to amino acid positions. **(B)** Subcellular localization of AFP2 truncations fused to full-length YFP in *N. benthamiana* pavement cells three days after infiltration (DAI). EV, empty vector control (full-length YFP). Scale bars, 20 μm. The merge channel shows overlay of the red (chlorophyll autofluorescence), differential interference contrast (DIC) and yellow (YFP fluorescence) channels.

AFPs have three conserved domains: the A domain, which contains the EAR domain; the B domain, which contains a putative nuclear localization signal found in AFP1, AFP2, and AFP3; and the C domain, which is necessary and sufficient for binding and repressing ABI5/ABF function (**Figure 2A**; Lopez-Molina et al., 2003; Garcia et al., 2008; Lynch et al., 2017). To better characterize the mechanism of CD induction by AFP2, we aimed to determine the domain required to induce CD. To this end, we constructed truncations of AFP2 based on sequence alignment to NINJA and other AFP proteins (Garcia et al., 2008, Pauwels et al., 2010; Lynch et al., 2017; **Figure 2A**). We then fused all AFP2 variants to full-length yellow fluorescent protein (YFP) to observe their subcellular localization in *N. benthamiana* epidermal cells and ensure stable expression.

When transiently expressed in *N. benthamiana* leaves, AFP2 was always localized to the nucleus (**Figure 2B**). This is in agreement with nuclear localization shown for other AFPs (AFP1 and AFP4) when transiently overexpressed in Arabidopsis mesophyll or onion epidermal cells (Lopez-Molina et al., 2003; Huang and Wu, 2007). However, in transgenic Arabidopsis plants expressing AFP2-GFP driven by the *AFP2* promoter, AFP2-GFP localizes to the nucleus only under conditions of stress or in the presence of ABA (Garcia et al., 2008). This suggests that the process of Agrobacterium–mediated infiltration may trigger a stress state in leaves. Alternatively, constitutive overexpression of AFP2 bypasses nuclear targeting regulation, leading to constitutive nuclear localization.

All YFP-AFP2 truncations were also found to localize to the nucleus, with AFP2_C_ showing the weakest YFP fluorescence. As expected, YFP alone was found in the cytoplasm and nuclei (**Figure 2B**). These results confirm previous studies showing that the B domain is not necessary for nuclear targeting, which suggests that there may be non-canonical nuclear localization sequences (NLS) in the EAR and C domain as well (Garcia et al., 2008; Lynch et al., 2017). Our localization studies also show that the YFP-AFP2 truncations are expressed in *N. benthamiana* and can be used in further studies.

### The EAR Domain Is Required for AFP2-Mediated Cell Death Induction in *N. benthamiana* Leaves

**Figure 3:**
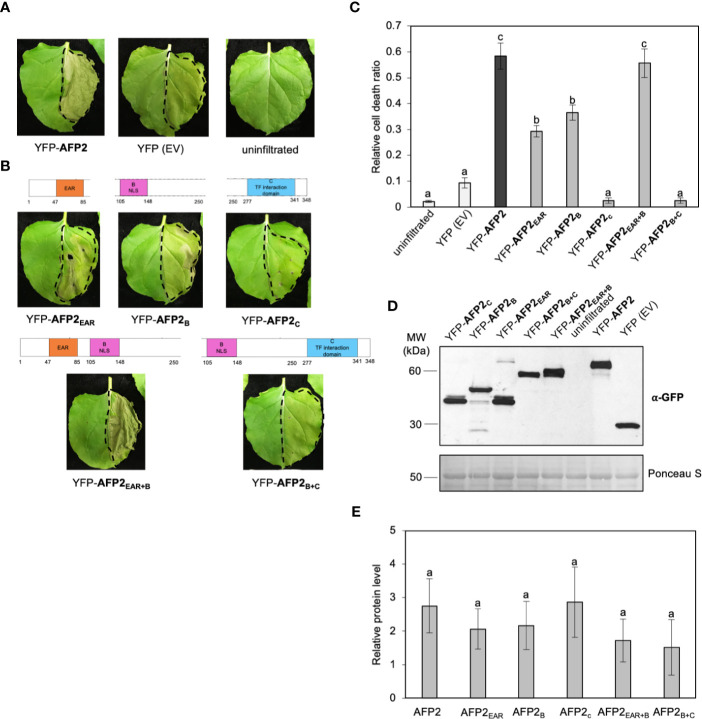
AFP2_EAR_ and AFP2_B_ domains are required for induction of PCD in *N. benthamiana*. **(A, B)** Cell death (CD) phenotype six days after infiltration (DAI) of AFP2 and its truncations fused to full-length YFP in *N. benthamiana* leaves. Dashed line delineates the infiltration area. **(C)** Relative quantification of cell death (dead tissue versus total infiltrated tissue) at six DAI using PIDIQ (Laflamme et al., 2016). Average of three biological replicates ± SEM is shown. p < 0.01, ANOVA and *post hoc* Tukey HSD test. **(D)** Immunoblot showing levels of AFP2 and its truncations three DAI in *N. benthamiana* leaf, using anti-GFP antibody. Ponceau stain is shown as loading control. **(E)** Graph showing relative quantification of AFP2 protein levels compared to the empty vector (YFP-EV) controls, which were set at 1. Values were normalized to the Rubisco large subunit as loading control. Average of three biological replicates ± SEM is shown. p > 0.1, ANOVA. Small letters represent the statistical grouping based on ANOVA and post hoc Tukey HSD test.

Previous research has shown that ethylene responsive TFs (ERFs) with EAR domain(s) from rice, tobacco and Arabidopsis can cause CD when ectopically overexpressed, and that the EAR domain is necessary but not sufficient for CD induction (Ogata et al., 2012; Cao et al., 2018). To determine if the EAR domain of AFP2 is required to induce CD, we quantified CD induced by the various AFP2 truncations. AFP2_EAR+B_-infiltrated leaves showed a strong CD phenotype, similar to full-length AFP2 (p < 0.01, ANOVA and *post hoc* Tukey HSD test; **Figures 3A–C**). AFP2_EAR_ and AFP2_B_ also induced CD, albeit with a milder effect (**Figures 3A–C**). No CD was observed when AFP2_C_ or AFP2_B+C_ were infiltrated, suggesting that EAR and B domains together are necessary and sufficient for the full induction of CD by AFP2.

Next, we examined whether the differences in CD induction were due to differences in protein levels of the AFP2 truncations. We performed Western blots on the same infiltrated tissue that was used for both subcellular localization (**Figure 2**) and quantification of the CD phenotype (**Figure 3**). All truncations were expressed at relatively similar levels (ANOVA p > 0.1), suggesting that induction of CD is due to the AFP2 variant infiltrated, and not the level of protein expression (**Figures 3D, E****)**.

### SnRK1α1 Interacts With AFP2_B_ and AFP2_C_ Domains *In Yeast* and *In Planta*

**Figure 4:**
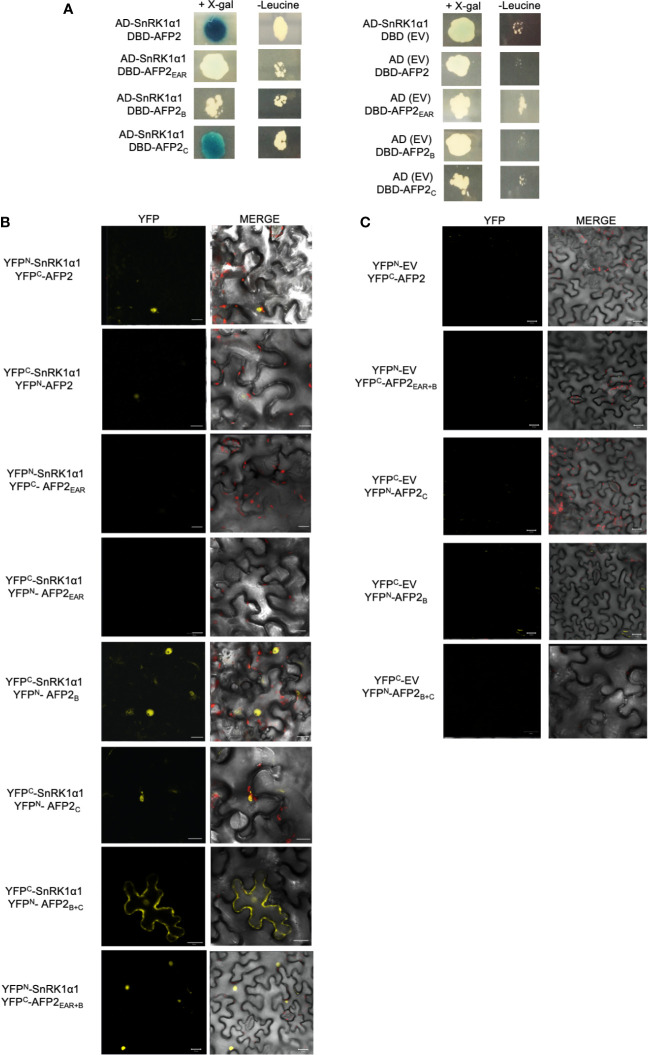
SnRK1α1 interacts with AFP2 B and C, but not EAR domain in yeast. **(A)** Yeast two-hybrid assay. AFP2 and its truncations were fused to the LexA DNA binding domain (DBD) and SnRK1α1 was fused to the B42 activation domain (AD). Pictures were taken four days after mating on plates containing X-gal (for *LacZ* reporter) or lacking leucine (for *LEU* reporter). Autoactivation of the reporters by SnRK1α1, AFP2 and its truncations was tested in the presence of the empty vector (EV; BD or AD) **(B)** Interaction between SnRK1α1 and AFP2 truncations fused to half-YFP (YFP^N^ or YFP^C^) by Bimolecular fluorescence complementation (BiFC) in *N. benthamiana* epidermal leaves. **(C)** BiFC controls of AFP2 truncations with the EV. Merge channel shows the DIC, YFP and chlorophyll autofluorescence (red). Scale bars are 20 μm.

Given the strong reduction of AFP2-induced CD by SnRK1α1, we focused on further characterizing the interaction between AFP2 and SnRK1α1. To identify the domain of AFP2 interacting with SnRK1, we conducted Y2H interaction assays using both the *LacZ* and leucine (*LEU*) reporter systems, in parallel. Yeast cells co-expressing SnRK1α1 with AFP2 or AFP2_C_ were able to activate both reporters, as shown by growth of yeast cells on – leucine plates and blue colour on X-gal plates, while yeast cells co-expressing SnRK1α1 with AFP2_B_ activated only the *LEU* reporter (**Figure 4A**). AFP2_EAR_ did not activate the *LacZ* reporter and results with the leucine reporter were inconclusive, since there was similar yeast growth also with the negative control (empty vector) (**Figure 4A**). SnRK1α1 and α2 autoactivated when fused to the DBD (Carianopol et al., 2020), therefore, reciprocal interactions could not be tested.

In order to confirm the Y2H results, we fused the AFP2 truncations to YFP^N^/YFP^C^ and performed BiFC in *N. benthamiana*. *In planta*, SnRK1α1 interacted with both AFP2_B_ and AFP2_C_ in the nuclei, but not with AFP2_EAR_, confirming the Y2H results (**Figure 4B**). SnRK1α1 also interacted with AFP2_EAR+B_ (**Figure 4**), which is necessary and sufficient for the full CD induction in *N. benthamiana* leaves (**Figure 3**). While the interactions of SnRK1 with full-length AFP2 was observed when proteins were fused to YFP^N^ or YFP^C^, interactions with the truncated AFP2 was observed primarily in one orientation (shown in **Figure 4**). Possibly, the small, truncated AFPs fused to the small, truncated YFP^N^/YFP^C^ affected protein folding and/or functionality depending on the orientation. Nevertheless, all interactions shown in **Figure 4** were observed in multiple cells per leaf and in multiple trials (at least in three separate experiments), with the exception of AFP2_EAR_ for which interaction with SnRK1could not be seen in neither orientation.

Together, this data suggests that SnRK1α1 interacts with several domains of AFP2, including the AFP2_EAR+B_ responsible for full CD induction.

### SnRK1α1 Antagonize AFP2_EAR+B_-Mediated Cell Death Induction

**Figure 5:**
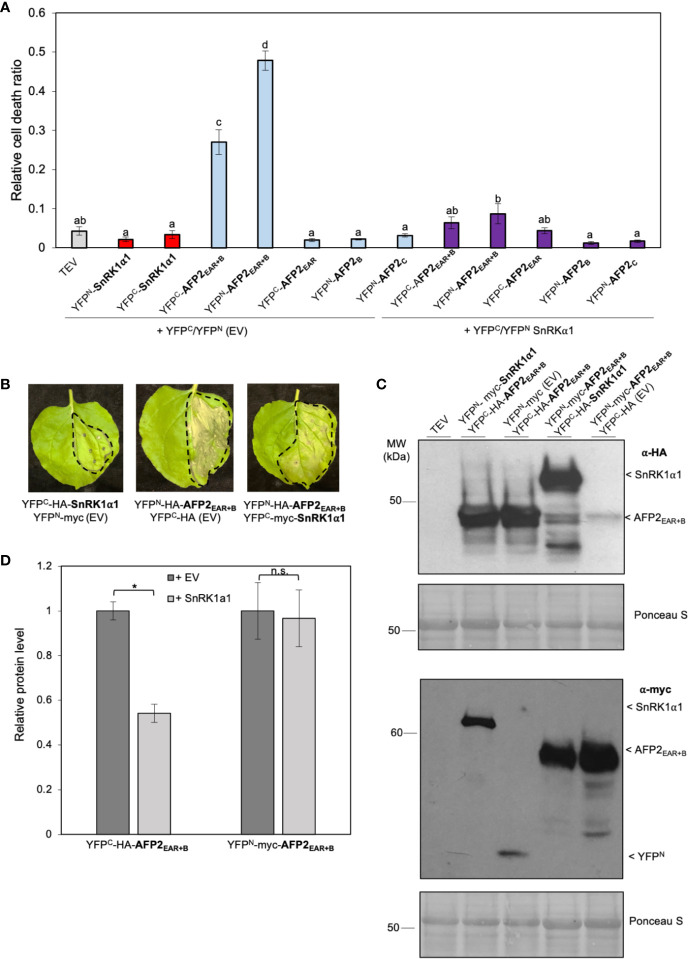
SnRK1α1 rescues AFP2_EAR+B_-induced PCD. **(A)** Relative quantification of cell death (dead tissue versus total infiltrated tissue) by ImageJ macro PIDIQ (Laflamme et al., 2016) six days after infiltration (DAI) of *N. benthamiana* leaves with various constructs fused to half-YFP. AFP2 or SnRK1α1 co-infiltrated with the empty vector (EV) carrying half-YFP (YFP^N^-myc or YFP^C^-HA) are shown as control. TEV, *N. benthamiana* leaves infiltrated with infiltration medium containing only TEV (infiltration control). Average of three biological replicates ± SEM is shown; p < 0.01, ANOVA and *post hoc* Tukey HSD test. **(B)** CD phenotype of *N. benthamiana* leaves six DAI. Dashed line delineates the infiltration area. **(C)** Immunoblot showing YFP^C^-HA-AFP2_EAR+B_ (37 kDa) and YFP^N^-myc-AFP2_EAR+B_ (45 kDa) protein levels in the presence of SnRK1α1 or EV in *N. benthamiana* leaves three DAI, using anti-HA or anti-myc antibody. Ponceau stain is shown as loading control. Results from one representative immunoblot of one biological replicate is shown. **(D)** Quantification of AFP2_EAR+B_ and SnRK1α1 protein level relative to the respective empty vector (EV) controls (protein level of YFP^C^-HA or YFP^N^-myc were set at 1). Values were normalized to the Rubisco large subunit for loading control. Average of three biological replicates ± SEM is shown; p < 0.05 (*), Student’s t-test. Small letters represent the statistical grouping based on ANOVA and post hoc Tukey HSD test.

To study the effect of SnRK1α1 on AFP2_EAR+B_ ability to induce CD, we monitored the development of CD in the same leaves that were infiltrated with the BiFC constructs (YFP^N^/YFP^C^ fusions) (**Figure 4**). As seen with the AFP2 truncations fused to full-length YFP (**Figure 3**), YFP^N^/YFP^C^-AFP2_EAR+B_ truncation had the highest induction of CD when co-infiltrated with the empty vectors (YFP^N^/YFP^C^-EV (**Figure 5A**). None of the other truncations showed CD level statistically above the control plants (p < 0.01; ANOVA and *post hoc* Tukey HSD test) (**Figure 5A**), although a mild induction of CD was observed when the AFP2_EAR_ and AFP2_B_ domains were fused to full-length YFP (**Figure 3**). One possibility is that fusion to the full length YFP may aid in proper folding of the small truncations compared to fusions to the half YFP. Interestingly, co-expression of AFP2_EAR+B_ with SnRK1α1 rescued the CD phenotype induced by AFP2_EAR+B_ alone (**Figures 5A, B****)**, suggesting that SnRK1α1 can antagonize AFP2_EAR+B_ induction of CD.

To determine if the rescue of AFP2_EAR+B_-induced CD by co-expression with SnRK1α1 was due to a difference in AFP2_EAR+B_ protein levels, we performed Western blots with proteins extracted from infiltrated *N. benthamiana* leaves used for CD quantification (**Figure 5A**). When co-expressed with YFP^N^-SnRK1α1, the relative protein levels of cYFP-AFP2_EAR+B_ was lower (Student’s t-test, p < 0.05) than when co-expressed with empty vector (YFP^N^ EV) control (**Figures 5C, D**). However, when switching the orientation of the half YFP fusions, the relative protein level of YFP^N^-AFP2_EAR+B_ did not change when co-expressed with YFP^C^-SnRK1α1 or the YFP^C^ (EV) control (**Figures 5C, D**). Notably, SnRK1α1 can rescue CD induced by both YFP^C^- and YFP^N^-fused AFP2_EAR+B_, regardless of the relative difference in YFP^N/C^-AFP2_EAR+B_ protein level (**Figure 5A**). This data suggests that the rescue of CD observed is not likely correlated to the expression level of the AFP2_EAR+B_ variant, but to the expression of and possibly interaction with SnRK1α1.

**Figure 6:**
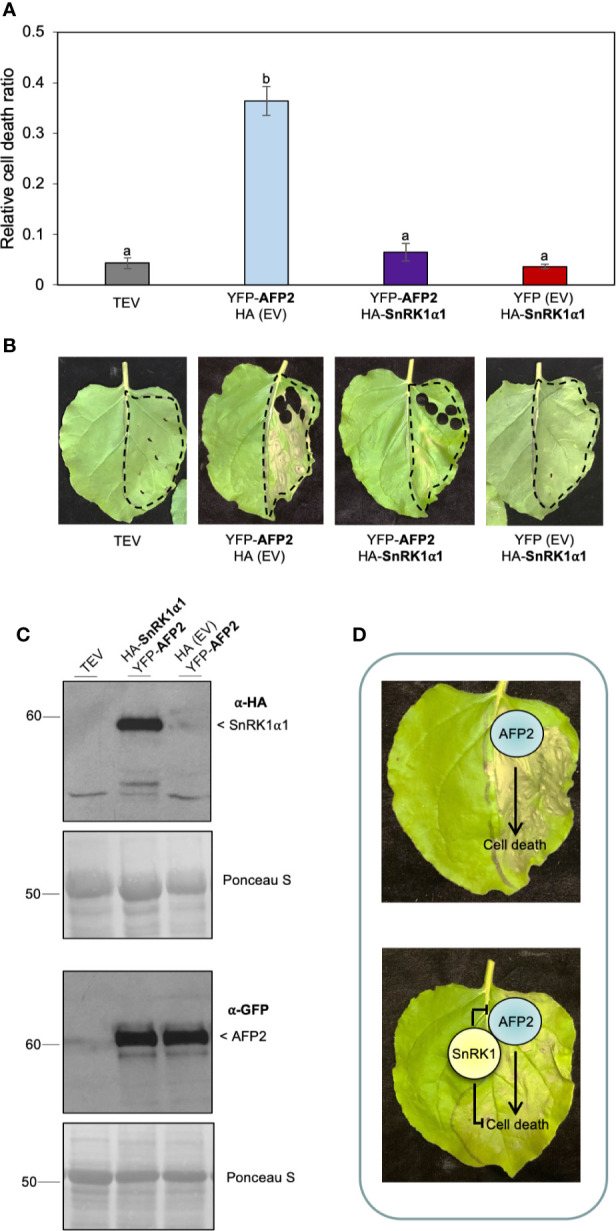
HA-SnRK1α1 rescues YFP-AFP2-induced PCD. **(A)** Relative quantification of cell death (dead tissue versus total infiltrated tissue) using ImageJ macro PIDIQ (Laflamme et al., 2016) at six days after infiltration (DAI) of *N. benthamiana* leaves. AFP2 is N-terminally fused to full-length YFP (YFP-AFP2), while SnRK1α1 is N-terminally fused to HA (HA-SnRK1α1). AFP2 or SnRK1α1 co-infiltrated with the empty vector (EV) are shown as controls. TEV, *N. benthamiana* leaves infiltrated with infiltration medium containing TEV (infiltration control). Average of three biological replicates ± SEM is shown; p < 0.01, ANOVA and *post hoc* Tukey HSD test. **(B)** Representative images of CD phenotype six DAI of SnRK1α1 and AFP2. **(C)** Immunoblot confirming the presence of HA-SnRK1α1 protein in *N. benthamiana* leaves three DAI with YFP-AFP2 (66 kDa), using anti-HA and anti-GFP antibodies, respectively. Ponceau stain of Rubisco large subunit (56 kDa) is shown as loading control. Results from one representative immunoblot of one biological replicate is shown. **(D)** Model showing that transient overexpression of AFP2 in *N. benthamiana* induces CD. Transient co-expression of SnRK1α1 inhibits, directly or indirectly, AFP2-induced CD. Small letters represent the statistical grouping based on ANOVA and post hoc Tukey HSD test.

SnRK1α1 can rescue AFP2-induced CD in BiFC assays, however the reconstitution of the full-length YFP protein in the SnRK1α1-AFP2 BiFC interaction is irreversible (Kudla and Bock, 2016). Therefore, we sought to determine whether this irreversible interaction was preventing the development of CD phenotype through the sequestration of AFP2. To test this, we co-infiltrated *N. benthamiana* leaves with AFP2 fused to full-length YFP (YFP-AFP2) and SnRK1α1 fused to HA only (no YFP) (**Figures 6A, B**). We found that SnRK1α1 could rescue AFP2-induced CD, regardless of the tag used (**Figures 6A, B**). To confirm that HA-SnRK1α1 and YFP-AFP2 proteins were expressed, we performed Western blots on tissue sample obtained from leaves used for tracking the CD phenotype. We found that both proteins were expressed and that YFP-AFP2 protein levels were unaffected by co-infiltration with HA-SnRK1α1 (**Figure 6**), similarly to what we had seen with the half-YFP fusions (**Figures 3**, **5**).

Altogether, these data suggest that SnRK1α1 can counteract the induction of CD triggered by AFP2 by a mechanism that does not involve a reduction in AFP2 protein level (**Figure 6C**), nor a change in subcellular localization (compare **Figures 2B** and **4B**).

## Discussion

AFPs play important roles in plant development, ABA and stress responses in plants, and do so by repressing transcription through recruitment of HDACs at target loci as well as other yet unknown mechanisms (Tang et al., 2016; Lynch et al., 2017; Chang et al., 2018; Chang et al., 2019). Recently, we showed that AFP2 interacts with the α1 and α2 kinase subunits of SnRK1 (Carianopol et al., 2020), a well conserved ser/thr kinase complex that is activated in response to stress to restore cellular energy homeostasis. However, the function of this interaction was unknown. Here, we show that AFP2 and SnRK1 α1 play antagonistic roles in CD. Transient overexpression of Arabidopsis *AFP2* in *N. benthamiana* leaves induces CD, which is rescued by co-expression of Arabidopsis *SnRK1 α1*, suggesting that SnRK1α1 counteracts AFP2 function during CD (**Figure 6D**). The AFP2_EAR+B_ truncation containing the EAR domain, which has been shown to bind TPL and TPR family of repressors and recruit HDACs (Pauwels et al., 2010; Lynch et al., 2017; Chang et al., 2019), is necessary and sufficient for the induction of CD, suggesting AFP2-induced CD may require transcriptional repression of target genes. Unlike SnRK1α1, SnRK1α2 did not rescue AFP2-triggered CD, pointing to different roles for the two catalytic subunits in CD. Thus, our findings uncover novel and contrasting roles for AFP2 and SnRK1 in CD.

### Transient Overexpression of AFP2 Induces CD and Requires the EAR Domain for CD Induction

AFP2-induced CD, which requires the EAR domain, resembles the programmed cell death (PCD) triggered by overexpression of transcription factors containing the EAR domain and belonging to APETALA2 (AP2)/ethylene response factors (ERF) or zinc finger families (Ogata et al., 2012; Ogata et al., 2014; Ogata et al., 2015; Cao et al., 2018; Cui et al., 2018). Several EAR-containing ERFs from tobacco, rice and Arabidopsis were shown to cause an HR-like CD phenotype when transiently overexpressed in *N. benthamiana*, and ERF-induced PCD was dependent on the EAR domain (Ogata et al., 2012; Ogata et al., 2014; Ogata et al., 2015; Cao et al., 2018). However, the EAR domain alone was not sufficient to trigger PCD and other sequences within the ERF proteins were shown to play an important role in promoting PCD (Ogata et al., 2015). The Arabidopsis ERF8 and SRG1 were shown to induces PCD when overexpressed in Arabidopsis, aside from transient overexpressed in *N. benthamiana*, and both were shown to play a role in pathogen response (Cao et al., 2018; Cui et al., 2018).

In contrast to ERFs, AFPs and related family members (NINJA) do not bind DNA directly, instead, they interact with TF or with adaptor proteins that bind to TF. AFPs interact with ABI5 to attenuate ABA signaling and with CONSTANS to delay flowering time (Pauwels et al., 2010; Lynch et al., 2017; Chang et al., 2019). Although the mechanisms behind AFP2-induced CD is unknown, the protective role previously shown by ABA during CD suggests that AFP2 may trigger CD by attenuating or inhibiting ABA responses. In germinating seeds of barley and maize, the endosperm undergoes PCD, a process that is induced by gibberellins (GA) and ethylene, and antagonized by ABA (Bethke et al., 1999; Young and Gallie, 2000). In rice, ABA delays ethylene- and GA-promoted cell death in root epidermal cell under submergence (Steffens and Sauter, 2005). Furthermore, spontaneous CD lesions developed on the leaves of the ABA-deficient *Imm9150* rice mutant and could be rescued by ABA application, suggesting that CD was due to decreased ABA levels (Liao et al., 2018). This mutant also displayed excessive accumulation of H_2_O_2_ and increased disease resistance. All these studies show that ABA antagonizes ethylene- and GA-induced PCD. In light of these findings, we propose that the AFP2-induced CD could be dependent on AFP2 role as a negative regulator of the ABA response.

### Transient Overexpression of SnRK1α1 Rescues AFP2-Induced CD

Our results show that SnRK1α1, but not SnRK1α2, rescues AFP2-induced CD, suggesting a specific role for SnRK1α1 in CD. The SnRK1α1 and SnRK1α2 kinases have been previously shown to play different roles in flowering time, as well as specific roles during osmotic and salt stress responses (Williams et al., 2014; Carianopol et al., 2020). High-throughput yeast two-hybrid screens identified different partners for SnRK1α1 and SnRK1α2, suggesting that SnRK1 kinases are likely to regulate plant growth and stress responses by interacting with different target proteins (Carianopol et al., 2020).

The mechanism behind SnRK1α1-mediated rescue of AFP2-induced CD is currently unknown, however our data suggest that it does not involve a change in protein level or AFP2 subcellular localization. Indeed, AFP2 protein levels showed little changes in the presence or absence of SnRK1α1, and CD induced by AFP2 or AFP2_EAR+B_ overexpression was always rescued by co-expression of SnRK1α1. Furthermore, AFP2 or AFP2_EAR+B_, which are nuclear localized, interacted with SnRK1α1 in the nuclei. Interestingly, SnRK1α1 interaction with the AFP2_EAR+B_ domain, which is responsible for CD induction and also for interaction with TPL/TPR, suggests that SnRK1α1 may interfere with AFP2 recruitment of TPL/HDACs during CD. It remains to be determined whether SnRK1α1 inhibition of AFP2 function involves its phosphorylation. Although the conserved SnRK1 phosphorylation motif was not found in AFP2 (Carianopol et al., 2020), we cannot exclude that SnRK1α1 may phosphorylate AFP2 on a non-canonical or partial SnRK1 motif. Indeed, FUSCA3 was previously found to be phosphorylated by SnRK1α1 on a partial SnRK1 motif (Tsai and Gazzarrini, 2012). It is also possible that SnRK1α1 interaction with AFP2 results in AFP2 sequestration from the TPL/HDACs complex. SnRK1α1 may also antagonize AFP2 function indirectly, by regulating CD through a different pathway (**Figure 6D**).

PCD plays an important role in development, growth and response to stress in eukaryotes. New evidence suggests autophagy is involved in the regulation and outcome of both developmental and stress-related PCD (Kabbage et al., 2017). There are several reports that suggest a link between SnRK1 modulation of autophagy and its role in (a)biotic stress response (Hulsmans et al., 2016; Signorelli et al., 2019). SnRK1 can promote autophagy under abiotic stress in Arabidopsis (Soto-Burgos and Bassham, 2017; Pu et al., 2017). In mammals, the SnRK1 orthologue, AMPK, can play a proapoptotic or pro-survival role depending on type of tissue and stress exposure, although the role of AMPK is highly complex and controversial during this process (Villanueva-Paz et al., 2016). SnRK1 may play a similar role to AMPK, by maintaining CD balance to promote plant survival in plants overexpressing AFP2. Further studies are required to better understand if the CD phenotype induced by AFP2 is indeed PCD, and the mechanisms behind SnRK1α1 ability to rescue AFP2-induced CD.

## Data Availability Statement

The raw data supporting the conclusions of this article will be made available by the authors, without undue reservation.

## Author Contributions

SG and CC designed the experiments and wrote the manuscript. CC conducted all of the experiments.

## Funding

This work was supported by an NSERC (Natural Sciences and Engineering Research Council of Canada) grant to SG and OGS (Ontario Graduate Scholarship) to CC.

## Conflict of Interest

The authors declare that the research was conducted in the absence of any commercial or financial relationships that could be construed as a potential conflict of interest.

## References

[B1] Bethke P. C. Lonsdale J. E. Fath A. Jones R. L. (1999). Hormonally regulated programmed cell death in barley aleurone cells. Plant Cell 11, 1033–1046. 10.1105/tpc.11.6.1033 10368175 PMC144253

[B2] Bitrián M. Roodbarkelari F. Horváth M. Koncz C. (2011). BAC-recombineering for studying plant gene regulation: developmental control and cellular localization of SnRK1 kinase subunits. Plant J. 65, 829–842. 10.1111/j.1365-313X.2010.04462.x 21235649

[B3] Broeckx T. Hulsmans S. Rolland F. (2016). The plant energy sensor: evolutionary conservation and divergence of SnRK1 structure, regulation, and function. J. Exp. Bot. 67, 6215–6252. 10.1093/jxb/erw416 27856705

[B4] Cao F. Y. DeFalco T. A. Moeder W. Li B. Gong Y. Liu X. M. (2018). Arabidopsis ETHYLENE RESPONSE FACTOR 8 (ERF8) has dual functions in ABA signaling and immunity. BMC Plant Biol. 18, 211. 10.1186/s12870-018-1402-6 30261844 PMC6161326

[B5] Carianopol C. S. Chan A. L. Dong S. Provart N. J. Lumba S. Gazzarrini S. (2020). An abscisic acid-responsive protein interaction network for sucrose non-fermenting related kinase1 in abiotic stress response. Commun. Biol. 3, 135. 10.1038/s42003-020-0866-8 32218501 PMC7099082

[B6] Causier B. Ashworth M. Guo W. Davies B. (2012). The TOPLESS interactome: a framework for gene repression in Arabidopsis. Plant Phys. 158, 423–438. 10.1104/pp.111.186999 PMC3252085 22065421

[B7] Chang G. Wang C. Kong X. Chen Q. Yang Y. Hu X. (2018). AFP2 as the novel regulator breaks high-temperature-induced seeds secondary dormancy through ABI5 and SOM in Arabidopsis thaliana. Biochem. Biophys. Res. Commun. 501, 232–238. 10.1016/j.bbrc.2018.04.222 29723526

[B8] Chang G. Yang W. Zhang Q. Huang J. Yang Y. Hu X. (2019). ABI5-BINDING PROTEIN2 Coordinates CONSTANS to Delay Flowering by Recruiting the Transcriptional Corepressor TPR2. Plant Phys. 179, 477–490. 10.1104/pp.18.00865 PMC6426417 30514725

[B9] Chiu R. S. Pan S. Zhao R. Gazzarrini S. (2016). ABA-dependent inhibition of the ubiquitin proteasome system during germination at high temperature in Arabidopsis. Plant J. 88, 749–761. 2 27496613 10.1111/tpj.13293

[B10] Crepin N. Rolland F. (2019). SnRK1 activation, signaling, and networking for energy homeostasis. Curr. Opin. Plant Biol. 51, 29–36. 10.1016/j.pbi.2019.03.006 31030062

[B11] Cui B. Pan Q. Clarke D. Villarreal M. O. Umbreen S. Yuan B. (2018). S-nitrosylation of the zinc finger protein SRG1 regulates plant immunity. Nat. Commun. 9, 4226. 10.1038/s41467-018-06578-3 30315167 PMC6185907

[B12] Cutler S. R. Rodriguez P. L. Finkelstein R. R. Abrams S. R. (2010). Abscisic acid: emergence of a core signaling network. Annu. Rev. Plant Biol. 61, 651–679. 10.1146/annurev-arplant-042809-112122 20192755

[B13] Finkelstein R. R. (2013). Abscisic acid synthesis and response. Arabidopsis Book 11, e0166. 10.1199/tab.0166 24273463 PMC3833200

[B14] Fujii H. Verslues P. E. Zhu J. K. (2007). Identification of two protein kinases required for abscisic acid regulation of seed germination, root growth, and gene expression in Arabidopsis. Plant Cell 19, 485–494. 10.1105/tpc.106.048538 17307925 PMC1867333

[B15] Fujita Y. Nakashima K. Yoshida T. Katagiri T. Kidokoro S. Kanamori N. (2009). Three SnRK2 protein kinases are the main positive regulators of abscisic acid signaling in response to water stress in Arabidopsis. Plant Cell Physiol. 50, 2123–2132. 10.1093/pcp/pcp147 19880399

[B16] Furihata T. Maruyama K. Fujita Y. Umezawa T. Yoshida R. Shinozaki K. (2006). Abscisic acid-dependent multisite phosphorylation regulates the activity of a transcription activator AREB1. Proc. Natl. Acad. Sci. U. S. A. 103, 1988–1993. 10.1073/pnas.0505667103 16446457 PMC1413621

[B17] Garcia M. E. Lynch T. Peeters J. Snowden C. Finkelstein R. (2008). A small plant-specific protein family of ABI five binding proteins (AFPs) regulates stress response in germinating Arabidopsis seeds and seedlings. Plant Mol. Biol. 67, 643–658. 10.1007/s11103-008-9344-2 18484180

[B18] Huang M. D. Wu W. L. (2007). Overexpression of TMAC2, a novel negative regulator of abscisic acid and salinity responses, has pleiotropic effects in *Arabidopsis thaliana*. Plant Mol. Biol. 63, 557–569. 10.1007/s11103-006-9109-8 17195036

[B19] Hulsmans S. Rodriguez M. De Coninck B. Rolland F. (2016). The SnRK1 energy sensor in plant biotic interactions. Trends Plant Sci. 21, 648–661. 10.1016/j.tplants.2016.04.008 27156455

[B20] Kabbage M. Kessens R. Bartholomay L. C. Williams B. (2017). The life and death of a plant cell. Annu. Rev. Plant Biol. 68, 375–404. 10.1146/annurev-arplant-043015-111655 28125285

[B21] Kudla J. Bock R. (2016). Lighting the way to protein-protein interactions: recommendations on best practices for bimolecular fluorescence complementation analyses. Plant Cell 28, 1002–1008. 10.1105/tpc.16.00043 27099259 PMC4904677

[B22] Laflamme B. Middleton M. Lo T. Desveaux D. Guttman D. S. (2016). Image-based quantification of plant immunity and disease. Mol. Plant-Microbe Inter. 29, 919–924. 10.1094/MPMI-07-16-0129-TA 27996374

[B23] Lewis J. D. Abada W. Ma W. Guttman D. S. Desveaux D. (2008). The HopZ family of Pseudomonas syringae type III effectors require myristoylation for virulence and avirulence functions in *Arabidopsis thaliana*. J. Bacteriol. 190, 2880–2891. 10.1128/JB.01702-07 18263728 PMC2293245

[B24] Liao Y. Bai Q. Xu P. Wu T. Guo D. Peng Y. (2018). Mutation in Rice *Abscisic Acid2* Results in Cell Death, Enhanced Disease-Resistance, Altered Seed Dormancy and Development. Front. Plant Sci. 9, 405. 10.3389/fpls.2018.00405 29643863 PMC5882781

[B25] Liu X. Yang S. Zhao M. Luo M. Yu C. W. Chen C. Y. (2014). Transcriptional repression by histone deacetylases in plants. Molecular Plant 7, 764–772. 24658416 10.1093/mp/ssu033

[B26] Lopez-Molina L. Mongrand S. Kinoshita N. Chua N. H. (2003). AFP is a novel negative regulator of ABA signaling that promotes ABI5 protein degradation. Genes Dev. 17, 410–418. 10.1101/gad.1055803 12569131 PMC195991

[B27] Lumba S. Toh S. Handfield L. F. Swan M. Liu R. Youn J. Y. (2014). A Mesoscale Abscisic Acid Hormone Interactome Reveals a Dynamic Signaling Landscape in Arabidopsis. Dev. Cell. 29, 360–372. 10.1016/j.devcel.2014.04.004 24823379

[B28] Lynch T. J. Erickson B. J. Miller D. R. Finkelstein R. R. (2017). ABI5-binding proteins (AFPs) alter transcription of ABA-induced genes via a variety of interactions with chromatin modifiers. Plant Mol. Biol. 93, 403–418. 10.1007/s11103-016-0569-1 27942958

[B29] Martin-Arevalillo R. Nanao M. H. Larrieu A. Vinos-Poyo T. Mast D. Galvan-Ampudia C. (2017). Structure of the Arabidopsis TOPLESS corepressor provides insight into the evolution of transcriptional repression. PNAS 114, 8107–8112. 28698367 10.1073/pnas.1703054114 PMC5544296

[B30] Ogata T. Kida Y. Arai T. Kishi Y. Manago Y. Murai M. (2012). Overexpression of tobacco ethylene response factor NtERF3 gene and its homologues from tobacco and rice induces hypersensitive response-like cell death in tobacco. J. Gen. Plant Pathol. 78, 8–17. 10.1007/s10327-011-0355-5

[B31] Ogata T. Kida Y. Tochigi M. Matsushita Y. (2014). Analysis of the cell death-inducing ability of the ethylene response factors in group VIII of the AP2/ERF family. Plant Sci. 209, 12–23. 10.1016/j.plantsci.2013.04.003 23759099

[B32] Ogata T. Okada H. Kawaide H. Takahashi H. Seo S. Mitsuhara I. (2015). Involvement of Nt ERF 3 in the cell death signalling pathway mediated by SIPK/WIPK and WRKY 1 in tobacco plants. Plant Biol. 17, 962–972. 10.1111/plb.12349 25996234

[B33] Pauwels L. Barbero G. F. Geerinck J. Tilleman S. Grunewald W. Pérez A. C. (2010). NINJA connects the co-repressor TOPLESS to jasmonate signaling. Nature 464, 788–791. 10.1038/nature08854 20360743 PMC2849182

[B34] Pu Y. Soto-Burgos J. Bassham D. C. (2017). Regulation of autophagy through SnRK1 and TOR signaling pathways. Plant Signal. Behav. 12, 1204. 10.1080/15592324.2017.1395128 PMC5792129 29058995

[B35] Raghavendra A. S. Gonugunta V. K. Christmann A. Grill E. (2010). ABA perception and signalling. Trends Plant Sci. 15, 395–401. 10.1016/j.tplants.2010.04.006 20493758

[B36] Rodrigues A. Adamo M. Crozet P. Margalha L. Confraria A. Martinho C. (2013). ABI1 and PP2CA phosphatases are negative regulators of Snf1-related protein kinase 1 signaling in Arabidopsis. Plant Cell 25, 3871–3884. 10.1105/tpc.113.114066 24179127 PMC3877788

[B37] Signorelli S. Tarkowski Ł.P. Van den Ende W. Bassham D. C. (2019). Linking Autophagy to Abiotic and Biotic Stress Responses. Trends Plant Sci. 24, 413–430. 10.1016/j.tplants.2019.02.001 30824355 PMC6475611

[B38] Soto-Burgos J. Bassham D. C. (2017). SnRK1 activates autophagy via the TOR signaling pathway in *Arabidopsis thaliana*. PLoS One 12, e0182591. 10.1371/journal.pone.0182591 28783755 PMC5544219

[B39] Steffens B. Sauter M. (2005). Epidermal cell death in rice is regulated by ethylene, gibberellin, and abscisic acid. Plant Phys. 139, 713–721. 10.1104/pp.105.064469 PMC1255990 16169967

[B40] Tang N. Ma S. Zong W. Yang N. Lv Y. Yan C. (2016). MODD mediates deactivation and degradation of OsbZIP46 to negatively regulate aba signaling and drought resistance in rice. Plant Cell 28, 2161–2177. 10.1105/tpc.16.00171 27468891 PMC5059794

[B41] Tsai A. Y. L. Gazzarrini S. (2012). Overlapping and distinct roles of AKIN10 and FUSCA3 in ABA and sugar signaling during seed germination. Plant Signal. Behav. 7, 1238–1242. 10.4161/psb.21549 22902692 PMC3493403

[B42] Villanueva-Paz M. V. Cotán D. Maraver J. G. Oropesa-Ávila M. de la Mata M. Pavón A. D. (2016). “AMPK Regulation of Cell Growth, Apoptosis, Autophagy, and Bioenergetics,” in AMP-activated Protein Kinase (Cham: Springer International Publishing), 45–71.

[B43] Vishwakarma K. Upadhyay N. Kumar N. Yadav G. Singh J. Mishra R. K. (2017). Abscisic acid signaling and abiotic stress tolerance in plants: a review on current knowledge and future prospects. Front. Plant Sci. 8, 161. 10.3389/fpls.2017.00161 28265276 PMC5316533

[B44] Williams S. P. Rangarajan P. Donahue J. L. Hess J. E. Gillaspy G. E. (2014). Regulation of Sucrose non-Fermenting Related Kinase 1 genes in *Arabidopsis thaliana*. Front. Plant Sci. 5:324. 10.3389/fpls.2014.00324 25071807 PMC4090914

[B45] Wurzinger B. Nukarinen E. Nägele T. Weckwerth W. Teige M. (2018). The SnRK1 Kinase as Central Mediator of Energy Signaling between Different Organelles. Plant Physiol. 176, 1085–1094. 10.1104/pp.17.01404 29311271 PMC5813556

[B46] Yoshida T. Mogami J. Yamaguchi-Shinozaki K. (2015). Omics Approaches Toward Defining the Comprehensive Abscisic Acid Signaling Network in Plants. Plant Cell Physiol. 56, 1043–1052. 10.1093/pcp/pcv060 25917608

[B47] Yoshida T. Christmann A. Yamaguchi-Shinozaki K. Grill E. Fernie A. R. (2019). Revisiting the Basal Role of ABA - Roles Outside of Stress. Trends Plant Sci. 24, 625–635. 10.1016/j.tplants.2019.04.008 31153771

[B48] Young T. E. Gallie D. R. (2000). Regulation of programmed cell death in maize endosperm by abscisic acid. Plant Mol. Biol. 42, 397–414. 10.1023/A:1006333103342 10794539

[B49] Zhou X. Hao H. Zhang Y. Bai Y. Zhu W. Qin Y. (2015). SOS2-LIKE PROTEIN KINASE5, an SNF1-RELATED PROTEIN KINASE3-type protein kinase, is important for abscisic acid responses in Arabidopsis through phosphorylation of ABSCISIC ACID-INSENSITIVE5. Plant Physiol. 168, 659–676. 10.1104/pp.114.255455 25858916 PMC4453773

[B50] Zhu J.-K. (2016). Abiotic stress signaling and responses in plants. Cell 167, 313–324. 10.1016/j.cell.2016.08.029 27716505 PMC5104190

