# Supplementary figures and images for: SnRK1α1 Antagonizes Cell Death Induced by Transient Overexpression of Arabidopsis thaliana ABI5 Binding Protein 2 (AFP2)
Source: Front Plant Sci. 2020 Sep 29;11:582208. doi: 10.3389/fpls.2020.582208 (PMC7550686; doi:10.3389/fpls.2020.582208)

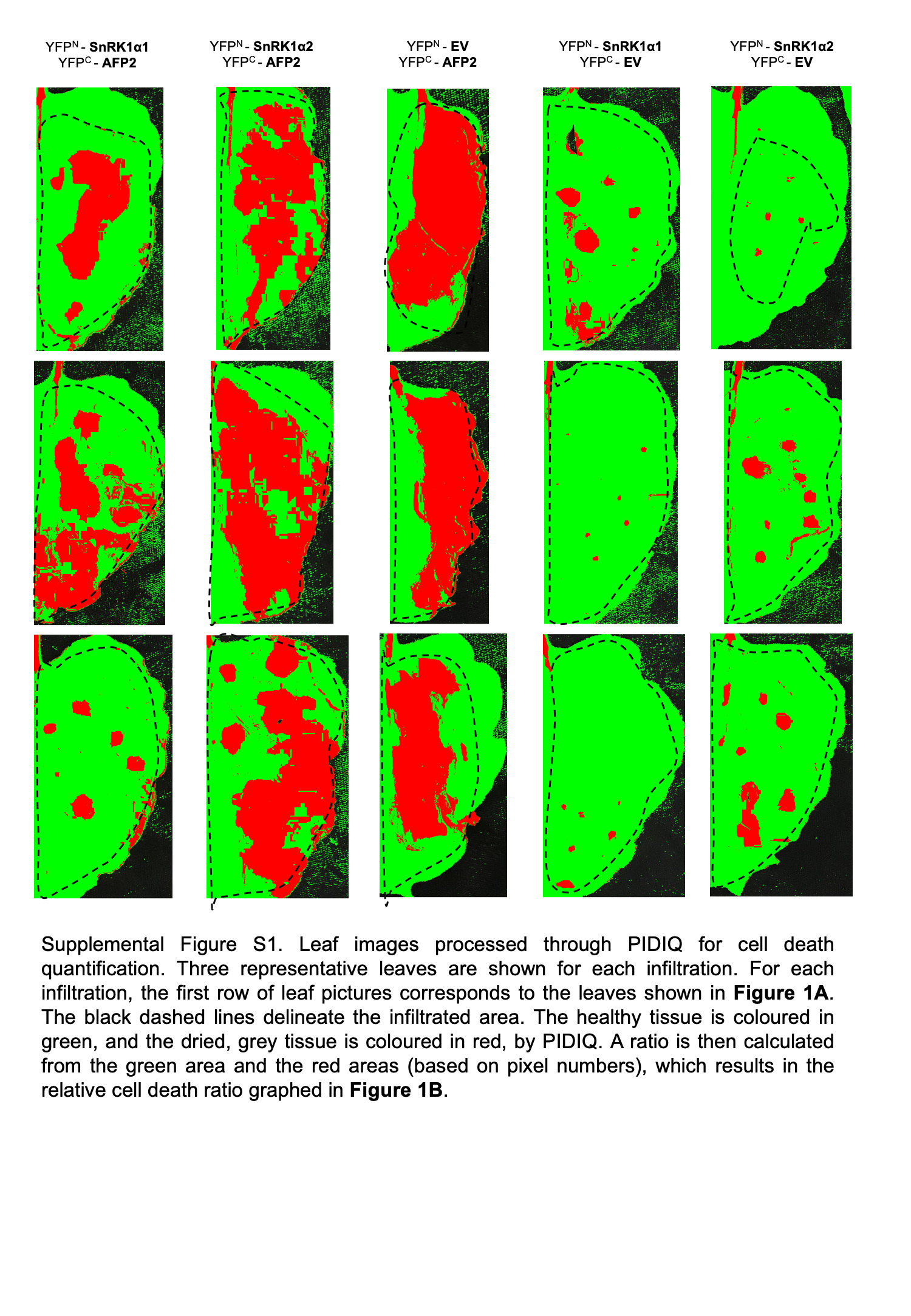

Supplement: Supplementary file 1 [file Image_1.tiff]
